# *De Novo* Transcriptome Sequencing in *Anopheles funestus* Using Illumina RNA-Seq Technology

**DOI:** 10.1371/journal.pone.0014202

**Published:** 2010-12-02

**Authors:** Jacob E. Crawford, Wamdaogo M. Guelbeogo, Antoine Sanou, Alphonse Traoré, Kenneth D. Vernick, N'Fale Sagnon, Brian P. Lazzaro

**Affiliations:** 1 Department of Entomology, Cornell University, Ithaca, New York, United States of America; 2 Centre National de Recherche et de Formation sur le Paludisme, Ouagadougou, Burkina Faso; 3 Department of Parasitology and Mycology, Centre National de la Recherche Scientifique Unit URA3012: Hosts, Vectors and Infectious Agents, Institut Pasteur, Paris, France; 4 Department of Microbiology, University of Minnesota, Saint Paul, Minnesota, United States of America; University of Montreal, Canada

## Abstract

**Background:**

*Anopheles funestus* is one of the primary vectors of human malaria, which causes a million deaths each year in sub-Saharan Africa. Few scientific resources are available to facilitate studies of this mosquito species and relatively little is known about its basic biology and evolution, making development and implementation of novel disease control efforts more difficult. The *An. funestus* genome has not been sequenced, so in order to facilitate genome-scale experimental biology, we have sequenced the adult female transcriptome of *An. funestus* from a newly founded colony in Burkina Faso, West Africa, using the Illumina GAIIx next generation sequencing platform.

**Methodology/Principal Findings:**

We assembled short Illumina reads *de novo* using a novel approach involving iterative *de novo* assemblies and “target-based” contig clustering. We then selected a conservative set of 15,527 contigs through comparisons to four Dipteran transcriptomes as well as multiple functional and conserved protein domain databases. Comparison to the *Anopheles gambiae* immune system identified 339 contigs as putative immune genes, thus identifying a large portion of the immune system that can form the basis for subsequent studies of this important malaria vector. We identified 5,434 1∶1 orthologues between *An. funestus* and *An. gambiae* and found that among these 1∶1 orthologues, the protein sequence of those with putative immune function were significantly more diverged than the transcriptome as a whole. Short read alignments to the contig set revealed almost 367,000 genetic polymorphisms segregating in the *An. funestus* colony and demonstrated the utility of the assembled transcriptome for use in RNA-seq based measurements of gene expression.

**Conclusions/Significance:**

We developed a pipeline that makes *de novo* transcriptome sequencing possible in virtually any organism at a very reasonable cost ($6,300 in sequencing costs in our case). We anticipate that our approach could be used to develop genomic resources in a diversity of systems for which full genome sequence is currently unavailable. Our *An. funestus* contig set and analytical results provide a valuable resource for future studies in this non-model, but epidemiologically critical, vector insect.

## Introduction

*Anopheles funestus* is a primary vector of human malaria parasites, which cause almost a million deaths of children under the age of 5 annually in sub-Saharan Africa [1]. The genome of *An. funestus* has not been sequenced, although it is expected to be within the next couple of years [2]. The current absence of a sequenced genome prevents many valuable experimental approaches from being applied to *An. funestus*, including determination of gene expression patterns after exposure to malaria parasites, comparison of genome content to other *Anopheles* and insect species, and reverse genetic manipulation to determine gene function. Despite the absence of a fully sequenced and assembled genome, however, many of these experiments could be pursued after sequencing of the transcriptome, the complete set of expressed genes.

Short read sequencing technologies such as the Solexa/Illumina (Illumina), 454 (Roche) and SOLiD (ABI) platforms have made it increasingly possible to perform *de novo* transcriptome sequencing [3]–[4]. For example, a single experiment on the instrument used in the present study (Illumina Genome Analyzer IIx, Illumina) can sequence 225–250 million nucleic acid molecules, generating 45–50 Gigabases of 100 base pair (bp) paired-end sequence in roughly 9.5 days, where “paired-end” refers to sequences obtained from the respective opposite ends of a single DNA molecule. As we will show, this volume of sequencing provides ample read coverage for *de novo* transcriptome assembly as well as for gene expression analyses and polymorphism discovery. The challenge with *de novo* transcriptome sequencing using data from short read technology lies in the difficulty of assembling the reads into contigs reflecting transcriptional units [3]. Short read sequence assembly is an active area of research, and has produced an array of assembly options (e.g. Velvet [5]; ALLPATHS2 [6]; ABySS [7]; Oases, Schulz and Zerbino, unpublished). The Roche 454 platform produces longer reads (∼450 bases) than the Illumina platform (<120 bp), helping to overcome the difficulties of *de novo* assembly, but Illumina produces orders of magnitude more sequence at a fraction of the cost, making it an attractive option for researchers with limited budgets. Despite this, *de novo* short read assembly of eukaryotic transcriptome sequence has been largely confined to 454-based sequencing efforts e.g. [8]–[10], with only a very few examples of *de novo* transcriptome sequencing using the Illumina platform occurring in the literature (e.g. *Pachycladon* [11]; Chinese Hamster Ovary Cell [12]; Whitefly [13]). Illumina-based transcriptome sequencing has been hampered in part by the absence of simple and effective assembly workflows capable of handling Illumina RNA-seq, or mRNA derived, datasets.

Understanding the basic biology of mosquito disease vectors such as *An. funestus* is essential for disease control efforts and development of new control technologies to be effective [14]. Valuable insights have been gained through studies of *An. gambiae* [15]–[16] and the sequencing of its genome [17], but *An*. *gambiae* is just one of several potent vectors of human malaria in Africa, and many open questions remain, including those regarding the genetic similarities and differences between the three most important and congeneric vectors: *An. gambiae, An. arabiensis* and *An. funestus*. *An. funestus* is estimated to have shared a common ancestor with the closely related sibling species pair *An. gambiae* and *An. arabiensis* 30–80 million years ago [18], and previous studies found the degree of genetic differentiation between *An. funestus* and *An. gambiae* to be high (substitutions per synonymous site (K_s_)  = 0.612±0.392 [19]), prohibiting simple use of the *An. gambiae* genome for gene discovery in *An. funestus* (such as through specific PCR in *An. funestus* with primers designed to the *An. gambiae* genome). Furthermore, *An. funestus* exhibits many epidemiologically important ecological differences from *An. gambiae*, including its ability to thrive in arid conditions unsuitable to many other vectors [20]–[21]. Disease control efforts will have to be tailored specifically to *An. funestus* in order to be fully effective. To date, there have been no efforts to sequence the complete transcriptome of *An. funestus*, although approximately 2,800 Expressed Sequence Tags (ESTs) have been obtained from traditional sequencing efforts aimed at genetic mapping [19], salivary gland protein discovery [22] and general transcript discovery [23].

We used the Illumina Genome Analyzer IIx platform (Illumina) coupled with a novel assembly approach to sequence the transcriptome of *An. funestus*. Historically, the generation of scientific data, genetic and otherwise, from *An. funestus* has been limited by the difficulty in rearing *An. funestus* in colony. We have recently established a new colony from specimens caught in Burkina Faso [Materials and Methods], bringing the number of *An. funestus* colonies worldwide from two [24] to three. We sequenced mRNA deriving from this colony using the Illumina sequencing platform and assembled the adult transcriptome of this species *de novo* using a hybrid assembly approach. Through bioinformatic analyses we identified ∼15,500 largely novel, high confidence transcription units. Short read alignments revealed almost 367,000 single nucleotide polymorphisms (SNPs) and insertion/deletion polymorphisms (indels), as well as substantial variation in expression levels among contigs. We confirmed homology of a large majority of our *An. funestus* contigs to several Dipteran transcriptomes, and identified 5,434 transcripts that could be paired to an *An. gambiae* gene as 1∶1 orthologues. Using bioinformatics, we putatively assigned contigs to broad functional categories and found that protein divergence was not evenly distributed among functional categories. Contigs that do not contain ambiguous bases or previously published *An*. *funestus* EST sequence have been deposited into NCBI and can be downloaded through the NCBI Sequence Read Archive website. The final catalog of our inferred transcriptional units is publicly available at www.jacobecrawford.com and at www.lazzaro.entomology.cornell.edu. We expect that this *An. funestus* transcriptome will provide a valuable genomic resource for future studies, including facilitating experimental genetic experiments and providing empirical support for gene models of the *An. funestus* genome when it is eventually sequenced.

## Materials and Methods

### *An. funestus* colony

We collected fed and gravid female *An. funestus* mosquitoes from the village of Koubri (12°11′54″N; 1°23′43″W) 35 kilometers South East of Ouagadougou, Burkina Faso, in February of 2007. Approximately 50 females were used to establish the colony, and the colony is maintained at a large size with overlapping generations in the insectary of Centre National de Recherche et de Formation sur le Paludisme in Ouagadougou, Burkina Faso. The females used to establish the colony were monomorphic with respect to chromosomal rearrangements and thus of the Kiribina chromosomal form as defined by [25]. We sampled females from the colony for mRNA extraction in November of 2008, corresponding to the 17^th^–19^th^ generation since the colony was established.

### RNA extraction and sequencing

3–5 day old female mosquitoes (n = 30) were removed from the colony, knocked down at −20°C, washed in ice-cold 95% ethanol to overcome the hydrophobic properties of mosquito cuticles and rinsed in ice-cold water, and then submerged in RNAlater (Qiagen, USA) and frozen at −80°C. Carcasses frozen in RNAlater were transported from Burkina Faso to the US where they were stored at −80°C. Total RNA was extracted using standard protocols (Trizol; Invitrogen, USA) from all 30 carcasses after grinding them under liquid nitrogen. mRNA selection, library preparation and sequencing was performed by the Cornell University Life Sciences Core Facilities on an Illumina GAIIx sequencer according to manufacturer specifications. Briefly, mRNA was selected using oligo(dT) probes and then fragmented using divalent cations. cDNA was synthesized using random primers, modified and enriched for attachment to the Illumina flowcell. We sequenced one 60-cycle paired-end lane and two 87-cycle paired-end lanes, generating ∼102.6 million reads for a total of 8,150 MB of sequence. All three un-filtered paired-end lanes of sequence have been deposited as a series with the accession number GSE21977 at NCBI's GEO database or at the NCBI Short Read Archive under submission number SRA020147.

### *De novo* transcriptome assembly

Prior to assembly and mapping (described below), we applied filters to remove low quality reads and reads containing suspected poly-Adenine tails from all three paired-end lanes. First, we implemented a ‘quality filter’ by removing reads where more than 33% of bases were ‘N’ and reads where more than 34% of the nucleotides had Phred quality scores less than 20, where a Phred score of 20 corresponds to a 1% expected error rate. Next, we removed sequences suspected of containing poly-Adenine tails by discarding any read composed of greater than 33% Adenine.

**Figure 1:**
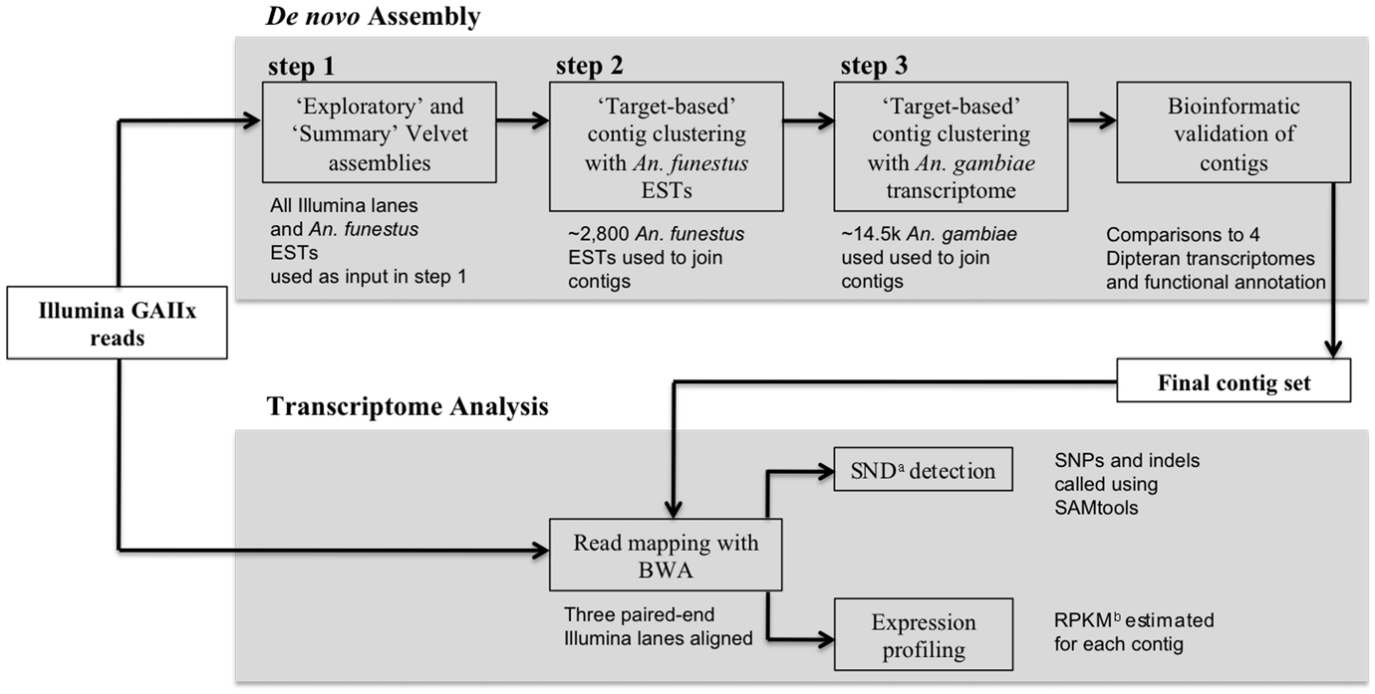
*De novo* transcriptome assembly and analysis workflow. Illumina reads were assembled in a series of ‘exploratory’ Velvet assemblies, the contig output of which was used in a ‘summary’ assembly. Following iterative assembly with Velvet, contigs were clustered and joined when possible, first using conspecific ESTs, then using the transcriptome of a closely related species. A final contig set was generated by selecting contigs based on bioinformatic support criteria. Illumina reads were then mapped to the final contig set and resulting alignments were used for expression profiling and polymorphism discovery. ^a^SND refers to short nucleotide discrepancies including both single nucleotide polymorphisms and indels. ^b^RPKM, or reads per kilobase per million mapped reads [40], was calculated for each contig and used to represent expression level.

Sequence assembly was carried out in three steps: 1) iterative *de novo* assembly with Velvet v7.58 [5], 2) ‘target-based’ clustering using *An. funestus* ESTs to find and unite, where possible, sequences belonging to the same transcription unit but not joined in Velvet, and 3) ‘target-based’ clustering using *An. gambiae* predicted peptides (Figure 1).

#### Step 1: Iterative Velvet assembly

For step 1, we used the *de novo* assembler Velvet to assemble all three lanes of paired-end Illumina reads. However, we implemented Velvet in a novel way in order to improve the assembly. First, we conducted ‘exploratory’ assemblies of the paired reads using multiple hash lengths (k = 21, 25, 31, 35, 41, 49, and 59). We then conducted an additional assembly (k = 57) of all unused reads (un-paired) from 4 of the exploratory assemblies (k = 21, 35, 49, 59). Next, we assembled all contigs obtained from all exploratory assemblies and the unused reads assembly in a series of ‘summary’ assemblies. First, we assembled all contigs at 3 different kmer values (k = 29, 39, 49) and then assembled the contigs obtained from these assemblies in a final summary assembly (k = 39). This “assembly of assemblies” approach may allow inclusion of some misassemblies, but these should result in low-confidence contigs that will be removed in subsequent steps in the workflow. Contigs from the final summary assembly were included in subsequent clustering steps.

#### Step 2: EST-based clustering

The highly coverage-sensitive nature of contig selection and node connection within the de Bruijn graph utilized by Velvet often results in partially fragmented assemblies. Therefore, we included the following clustering step to ensure all homologous sequence was joined where possible. For step 2, we downloaded all *An. funestus* ESTs from Genbank (n = 2,846 as of November 2009; referred to as “ESTs” below) [19] [22]–[23]. From this larger set of ESTs, We found 1,496 unique ESTs and used this condensed set as targets in a ‘target-based’ clustering process in order to join homologous contigs that were not joined in the Velvet assembly. We first used BLASTN from the stand-alone bundle of BLAST algorithms v2.2.23+ [26] to identify all contigs that showed significant similarity (e-value≤1×10^−6^) with each *An. funestus* EST downloaded from Genbank. Each matching contig was then individually aligned to its EST match using ClustalW [27], and contig-EST matches were discarded if their ClustalW alignment score was not greater than 50 plus 3 times the length of the shorter of the two sequences. All remaining contigs were then grouped by their matching EST and compared to the match with the highest BLAST score by dividing all BLAST scores by the maximum score in the group. Contigs with normalized BLAST scores less than 0.7 were discarded from further clustering steps. If more than one contig remained in an EST-group after the two previous filtering steps, contigs within each EST-group were aligned in a global alignment using ClustalW. To identify good matches between the EST and individual contigs, individual pairwise alignment scores for each EST-contig alignment within the global alignment were divided by the maximum EST-contig alignment score in that group, and all contigs with a normalized pairwise alignment score less than 0.7 were eliminated from further clustering. The cutoff value of 0.7 used in the previous filtering steps was chosen after visual inspection of a subset of alignments suggested that this criteria readily distinguished credible matches from those more likely to be spurious. Contigs that survived all of these filtering steps were then aligned to their EST match, and any sequence that extended further than the edge of the EST was joined to the EST and the total sequence was used in the final contig set. After this clustering process, the resulting contig set contained some contigs that were comprised partly of contig sequence and partly of EST sequence, some contigs that were comprised of two contigs joined in the middle by EST derived sequence, some contigs that resulted from joining two Velvet contigs and many sequences that were unaffected by the clustering process. Clustering and joining of contigs was accomplished with custom scripts written in the statistical computing environment R [28].

#### Step 3: *An. gambiae-*based clustering

For the final assembly step, step 3, we used the *An. gambiae* predicted peptide set (release 3.5) downloaded from Vectorbase.org in a second and analogous ‘target-based’ clustering step. The clustering step based on *An. funestus* ESTs (step 2 above) was helpful, but was not likely to be exhaustive due to the limited number of *An. funestus* ESTs available in public databases, so we performed the following additional clustering step using the much more complete, albeit evolutionarily diverged, *An. gambiae* transcriptome. All contigs that were not joined in the EST-based clustering in step 2 were evaluated in analogous fashion against *An. gambiae* peptides. These contigs were compared to the entire *An. gambiae* predicted peptide set using BLASTX and submitted to the same filtering step as in the EST-clustering step above, where contigs with a normalized BLAST score less than 0.7 were disregarded. Surviving contigs were then grouped based on their peptide match. If more than two contigs matched a peptide, they were globally aligned using ClustalW. Pairwise ClustalW alignment scores within the global alignment of greater than 80 were considered positive matches and these contigs were joined. If only two contigs matched a peptide, they were aligned and if they overlapped with an alignment score greater than 90, they were joined into a single contig. If the contigs did not overlap in alignment, they were joined together by a string of ‘N’s using the peptide BLAST high scoring pair coordinates of each contig as a guide for the length of the N string. Like the EST-based clustering step, this clustering process was performed using custom R scripts.

At the end of step 3 of the assembly pipeline, the total contig set was comprised of many contigs that were not affected by the clustering process, some contigs that were the product of one or two contigs having been concatenated with pre-existing EST sequence, some contigs that were joined during the *An. gambiae* peptide clustering step, and some contigs that had been scaffolded around a run of ‘N’s.

### Bioinformatics and contig validation

To distinguish between valid transcript sequence and spuriously assembled sequence we compared the post-clustering set of contigs to multiple Dipteran insect transcriptomes, searched for open reading frames and compared translated protein sequences to functional protein domain databases as a means to identify contigs with bioinformatic associations with other species. First, we searched our assembled and clustered *An. funestus* contigs for homology to the translated predicted transcriptomes of other Dipteran insects with sequenced genomes. In addition to the *An. gambiae* peptide set used for clustering above, we downloaded the predicted peptide set from *Aedes aegypti* (release 1.2) and *Culex quinquefasciatus* (release 1.2) as well as the full genome sequence of *An. gambiae* (release 3) from Vectorbase.org. We also downloaded the predicted peptide set of *Drosophila melanogaster* (release 5.26) from Flybase.org. For reference, the genus *Anopheles* (Subfamily Anophelinae) is predicted to have shared a common ancestor with *Aedes* and *Culex* (Subfamily Culicinae), between 145–200 million years ago [18] and a common ancestor with *Drosophila* 260 million years ago [29]. We compared our final contig set to each of these four translated transcriptomes using BLASTX, as well as to the *An. gambiae* genome using TBLASTX, and high scoring matches with a minimum e-value of 1×10^−6^ were kept for further analysis. As part of functional annotation (described below), we also compared our contig set to the nr database at NCBI as the first step of Gene Ontology [30] (hereafter referred to as GO) annotation implemented by Blast2GO [31] using an expect value cutoff of 1×10^−6^.

In addition, we evaluated the post-clustering contig set based the size of the inferred open reading frame (ORF) relative to contig size. We extracted open reading frames from all contigs using the ‘-getorf’ function in the EMBOSS package [32]. To accommodate the uncertainty of whether our contig captured the full ORF, we extracted both translated regions that were flanked by a Methionine and a STOP codon (‘-find 1’; hereafter type A) as well as translated regions that were simply free of STOP codons (‘-find 0’; hereafter type B). For each contig, we compared the largest ORF from each of these types and kept the ORF that contained a start codon unless the type B ORF extended upstream of the type A ORF to the beginning of the contig representing cases in which the true start codon is likely truncated from the contig. If no type A ORF was found, the type B ORF was chosen. Then, in order to cleanse the contig set of contigs comprised of spuriously assembled sequence, we discarded any contig if its ORF was shorter than 50 amino acids.

To identify putative conserved protein domains and assign putative functional information to the post-clustering contig set, we compared translated protein sequences extracted from our contigs to multiple functional domain databases using RPS-BLAST and Blast2GO [31]. First, the total peptide set was compared to the SMART [33], KOG [34], Pfam [35] and CDD [36] databases using RPS-BLAST with no expect value threshold cutoff, but only matches with an expect value less than 1×10^−6^ were considered in further analyses. We also mapped our contigs to the GO database using Blast2GO. Annotation through Blast2GO is accomplished by first searching for matches to the nr database at NCBI, then mapping to the BLAST results to the GO database and finally selecting a GO annotation using their Annotation Rule that is based on the degree of similarity to the GO, GO Evidence Code weights (default values used here) and relative weights given to child versus parent terms [31]. In order to simplify the functional annotations to a set of broad terms, we also mapped the GO annotations to the Generic GO-Slim terms using Blast2GO. All results from BLAST comparisons to functional and conserved protein domain databases as well as the GO annotations are presented in Table S1.

We chose a final contig set by comparing results of all of the BLAST and functional domain database comparisons and keeping only sequences that showed a significant association to at least one proteome or database. This resulted in a conservative set of contigs, although it prevents the discovery of novel genes in the *An. funestus* transcriptome. This is an unfortunate consequence of the inherent difficulty in distinguishing novelties from spuriously assembled sequence. Our contig set as reported is composed entirely of high confidence transcription units. All contigs that did not contain any ‘N’s inserted during contig clustering (n = 14,980) are available in the Transcriptome Assembly Archive at NCBI under the accession numbers EZ966136 - EZ980985. The full final contig set is available at www.jacobecrawford.com and www.lazzaro.entomology.cornell.edu.

After compiling a conservative set of contigs using the bioinformatic and ORF filtering criteria, we performed a reciprocal best-hit analysis to identify 1∶1 orthologues between our *An. funestus* contigs and *An. gambiae* predicted proteins. First, we searched the *An. gambiae* peptide set with BLASTX using *An. funestus* contigs as queries with an e-value threshold of 1×10^−6^. We then performed the reciprocal search with TBLASTN using *An. gambiae* peptides as the queries and the same e-value threshold. *An. gambiae* peptides shorter than 50 amino acids (n = 26) were omitted from this search because the BLAST algorithm is unable to parse such short sequences. One-directional ‘best-hits’ were declared for each query if only a single BLAST result was obtained or the ratio of the BLAST score of the ‘second-best-hit’ to the BLAST score of the first ‘best-hit’ was less than 0.7. One-directional ‘best-hits’ were identified in both directions and 5,434 reciprocal ‘best-hits’ were obtained by comparing these datasets.

### Read mapping, SND calling and expression profiling

We used the short read alignment algorithm BWA [37] to align all three paired-end lanes of Illumina sequence reads to the final contig set established above. Prior to the assembly steps, all sequence reads were screened for low quality and low complexity as described above. To accommodate the global mapping procedure used in BWA and reduce the number of reads not mapped because of sequencing errors in the terminal end of the read, positions 76–87 of all remaining reads in the two lanes of 87 bp paired-end reads were trimmed using the FastX toolkit [http://hannonlab.cshl.edu/fastx_toolkit/], leaving 75 bp reads for mapping. The trimmed 75 bp and 60 bp paired-end reads were then aligned to the reference final contig set in BWA, with the maximum number of difference between each read and reference sequence set to 5 (‘aln -n 5’). Alignment files from the three paired-end lanes were merged, sorted and parsed by contig identification using pileup in the SAMtools package [38]. The consensus base, putative single nucleotide polymorphisms and short indels were called using the pileup ‘-c’ option. We called single nucleotide polymorphisms and indels (hereafter collectively referred to as short nucleotide discrepancies or SNDs) at sites where 1) the mapping quality was greater than or equal to 20, 2) the alternative base occurred at least twice or the equivalent of 0.025 times the coverage at the site when coverage was greater than 80, 3) only one alternative base occurred at or above this frequency and 4) at least 6 reads covered the site. Thus, in order to be considered a putative SNP, the alternative nucleotide would have to be observed with high confidence at least twice even at a positions covered by 6 reads. In this way, we aimed to decrease false positive SND calls from sequencing errors, which are generally expected to be unique in the read set, but which should accumulate in abundance linearly with sequence depth.

We were interested in determining colony-level genetic variation, recognizing that because the polymorphisms reported here were obtained from a colony of mosquitoes and not a random population sample, true population genetic parameters describing the natural population can not be appropriately estimated from this data. Estimates of genetic variation from high-throughput sequencing data are complicated by the fact that read depth varies among and within contigs and that highly expressed genes are more likely to be completely sequenced and thus represented by more bases. While raw SND counts are presented for the purpose of SND discovery, we applied several corrections and assigned each contig an adjusted nucleotide diversity (hereafter simply referred to as nucleotide diversity) value. First, we treated any bases that were not covered by at least 6 reads as missing data, so we calculated an initial estimate of nucleotide diversity by dividing all SND counts by the number of bases across the contig that were covered by at least 6 reads to obtain an estimate of SNDs per base. Next, to control for ascertainment bias related to variable read depth, or in this case expression level and mapping success, we adjusted length-corrected SND counts using the read-depth correction (eq. 7) proposed by Jiang et al. [39] that accounts for the possibility of missing data at low coverage sites and the probability of observing a mutant allele in a given sample. This correction is intended for regions of a genome with identical read depth [39], but since this requirement is not applicable to our case, we used the median read coverage per contig.

We were also interested in testing the utility of the assembled transcriptome for measuring gene expression. To estimate mapping success, we quantified the total number of reads mapped and further distinguished between uniquely mapped reads and repetitively mapped reads. We also quantified gene expression in our dataset extracting the number of reads mapped to each contig during the BWA alignment. However, Gene expression levels can be estimated from RNA-seq data with great accuracy e.g. [40], but, since read mapping is sensitive to the size of the target reference sequence, corrections must be applied to adjust for contig length. Therefore, we adjusted the raw read count by the total number of reads mapped and the length of the contig, calculating Reads Per Kilobase per Million mapped reads (RPKM; [40]).

### Protein divergence

To identify functional categories of proteins that show high levels of divergence or conservation, we determined protein divergence between 1∶1 *An. funestus*:*An. gambiae* orthologues. First, we aligned orthologous protein sequences using ClustalW. We then calculated protein distance using the ‘identity’ mode of the dist.alignment function in the R package seqinr [41]. This function calculates protein distance as the square root of the proportion of the sequence that is different between the two sequences. However, automated sequence alignment is unreliable at high divergence levels, so we excluded orthologous pairs with less than 30% identity (leaving 4,975 contigs) to avoid false mismatches introduced by low confidence alignments. Lastly, we assigned each orthologous pair to one of three categories based on its level of divergence (or proportion amino acids that differ): High (≥0.138), Intermediate (<0.138 and >0.058) and Low (≤0.058) divergence, with bin cutoffs empirically determined so that one-third of transcripts fall into each category.

### Comparison among functional categories

We used *Χ* ^2^ analyses to ask whether any functional categories of contigs as assigned above were significantly enriched or depleted of any of the nucleotide diversity categories. As described above, we first assigned contigs to a protein divergence bin (i.e. Low, Intermediate or High), and then to functional categories based on GO-Slim terms. We then, using a *Χ* ^2^ test, asked whether each GO-Slim category was enriched (or depleted) for any of the bins compared to the expectation of equal proportions expected under the binning method. We used a Bonferroni-adjusted α level of 5.26×10^−4^ to assign significance in tests. As the power of *Χ* ^2^ analyses increases with increasing number of observations, we limited our intra-functional category comparisons to categories populated by at least 15 contigs.

### Distribution of Data and Scripts for Analysis

An Excel spreadsheet, modeled after AnoXcel, containing peptide sequences, BLAST results, functional annotation results and other pertinent information for each contig in the final contig set is available online as Table S1 as well as on the websites www.jacobecrawford.com and www.lazzaro.entomology.cornell.edu. All data and results management, manipulations and analyses were carried out using custom scripts written by J. Crawford in the statistical computing environment R unless specified otherwise. Additionally, a Velvet wrapper script written in python by J. Crawford called AssemblyAssembler.py to automate the iterative Velvet assembly used here is available at the websites given above and is also packaged with Velvet, starting with version 0.7.63.

## Results and Discussion

### Sequencing and Assembly

**Figure 2:**
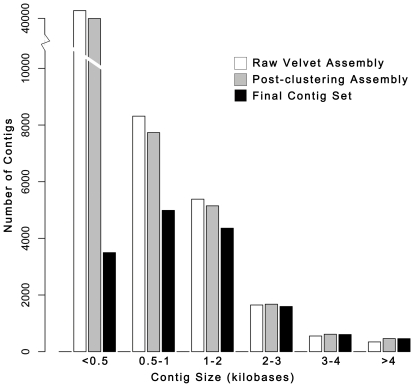
Size distribution of contigs at three points of the assembly. Note that the y-axis is broken between 10,000 and 40,000. White bars indicate the size distribution of contigs generated by the iterative Velvet assembly. Grey bars indicated the size distribution of contigs after ‘target-based’ clustering to both *An. funestus* ESTs and *An. gambiae* peptides. Black bars indicate the size distribution of the final contig set after quality filtering and bioinformatic analysis. The final contig set contains 15,527 contigs with an N50 of 1,753 bp.

Due to the low cost and ability to obtain both novel sequence for assembly as well as gene expression data, there is great interest in utilizing Illumina RNA-seq data for *de novo* transcriptome assembly and analysis [3]. We sequenced three paired-end lanes of mRNA extracted from 30 whole, sugar-fed female *An. funestus* using the Illumina Genome Analyzer. Approximately 102 million reads (or 51 million paired-end reads) passed Illumina quality filtering totaling roughly 8.1 GB of sequence. We removed 2% of these reads flagged as either low-quality or low-complexity. A first pass Velvet assembly with default parameters and a hash length of 31 yielded over 440,000 contigs and an N50 of 209 bp (i.e. 50% of the total assembled sequence was contained in contigs of this length or longer). We searched a large range of hash values (k = 21 to k = 59) and obtained a slight improvement by setting the hash value to 57, producing approximately 357,000 contigs with an N50 of 228 bp. Further exploration of various parameter settings and data combinations suggested that an iterative assembly in which contigs output from generic Velvet assemblies using various hash lengths are assembled in a final series of Velvet runs produced the best assembly (Figures 1 and 2). This final contig set of 46,987 contigs with an N50 of 1,140 bp comprised 27.8 MB of sequence and was submitted to further downstream filtering and analysis as detailed in Materials and Methods and briefly described below.

While the present manuscript was in review, several independent efforts to optimize transcriptome assembly using RNA-seq data were made publicly available. We were encouraged by the results of one independent study that obtained high quality transcriptome assemblies of Illumina reads using an iterative, varied kmer approach similar, in principle, to ours [42]. Two other efforts that employ an alternative approach have also been made available (Oases [Schulz and Zerbino, unpublished] and Cufflinks [43]). To determine how the performance our method compares to an alternative method, we assembled the *An. funestus* transcriptome using Oases with standard parameter settings and obtained a high quality assembly. This approach generated approximately twice as many contigs as our pre-clustering contig set, but the contig sets were very similar with respect to the proportion of sequences showing homology to *An. gambiae* and the N50 value. However, one key difference is that Oases relies heavily node scaffolding using paired-end information (74.8% of contigs contain ‘N’s in Oases contig set generated here), which is not ideal because these ambiguous bases produce ‘edge-effects’ in short-read mapping analyses.

In principle, the iterative assembly routine employed here is intended overcome the heterogenous coverage distribution inherent to non-normalized RNA-seq data by taking advantage of the fact that some contigs will be assembled best in certain assembly conditions while others are best assemble in different conditions. We and a colleague found anecdotal evidence using independent datasets that high coverage contigs assemble best in high kmer value assemblies, while low coverage contigs assemble best in low kmer value assemblies. Further exploration is needed to determine whether this can be exploited more directly. Importantly, we subsampled our data and found that this assembly routine produced a very respectable assembly (maximum contig length = 12,688 bp and N50 = 784 bp) with only single paired-end lane of Illumina sequence reads, suggesting that significant progress can be made with very little sequencing cost.

Following the iterative assembly step, we used ‘target-based’ clustering to improve the *de novo* assembly. By clustering contigs around previously described *An. funestus* ESTs and then predicted *An. gambiae* peptides, we searched the contig set for potential overlaps and joined contigs where possible and appropriate. This ‘target-based’ contig clustering process resulted in only a modest condensation of the contig set from 46,987 contigs to 45,644 contigs, a 2.9% reduction (Figure 2). In their Illumina-based assembly of the transcriptome of Chinese hamster ovary cells, Birzele et al. [12] utilized a similar assembly workflow that was a hybrid of *de novo* assembly and read mapping to the phylogenetically closest sequenced model system genome to improve assembly and annotation and achieved similarly modest assembly improvements [12]. Birzele et al. [12] used transcripts from the closely related mouse genome to cluster short reads for assembly leading to a reduction in their contig set of approximately 6% to 92,272 contigs with a mean length of 352 bp. Our own experience and the report of Birzele et al [12] suggests that, in general, clustering may not be an extremely effective means of improving *de novo* transcriptome assemblies. In contrast, our pre-clustering iterative assembly process generated 46,987 contigs with a mean length of 591 bp, underscoring the potential gains to be made through alternative *de novo* assembly approaches even in the absence of any clustering.

To purge our contig set of spuriously assembled sequence, we utilized bioinformatic support to validate our contigs. We eliminated any contig that did not show a significant BLAST match to at least one of four insect transcriptomes or functional databases and did not harbor a convincing ORF (Materials and Methods), leaving 15,527 contigs with an N50 of 1,753 bp (Figure 2). For comparison, the predicted transcript sets of the most thoroughly annotated Dipteran genomes, *An. gambiae* and *D. melanogaster*, are comprised of 14,753 and 21,921 transcripts, respectively, with N50s of 2,258 and 2,475 bp, respectively. This suggests that, not surprisingly, our contigs are frequently incomplete and represent only a subset of the potentially expressed transcriptome. We restricted our final contig set to a limited number of conservative contigs, reducing the total number of contigs relative to other *de novo* transcriptome studies e.g. [9] [12]. Even so, our high confidence contig set of 15,527 transcripts represents a marked expansion of the *An. funestus* genetic sequence space over the previously available ∼2,800 ESTs (521 of which we were able to extend through our assembly and clustering process).

### Homology with Dipteran sequences

**Figure 3:**
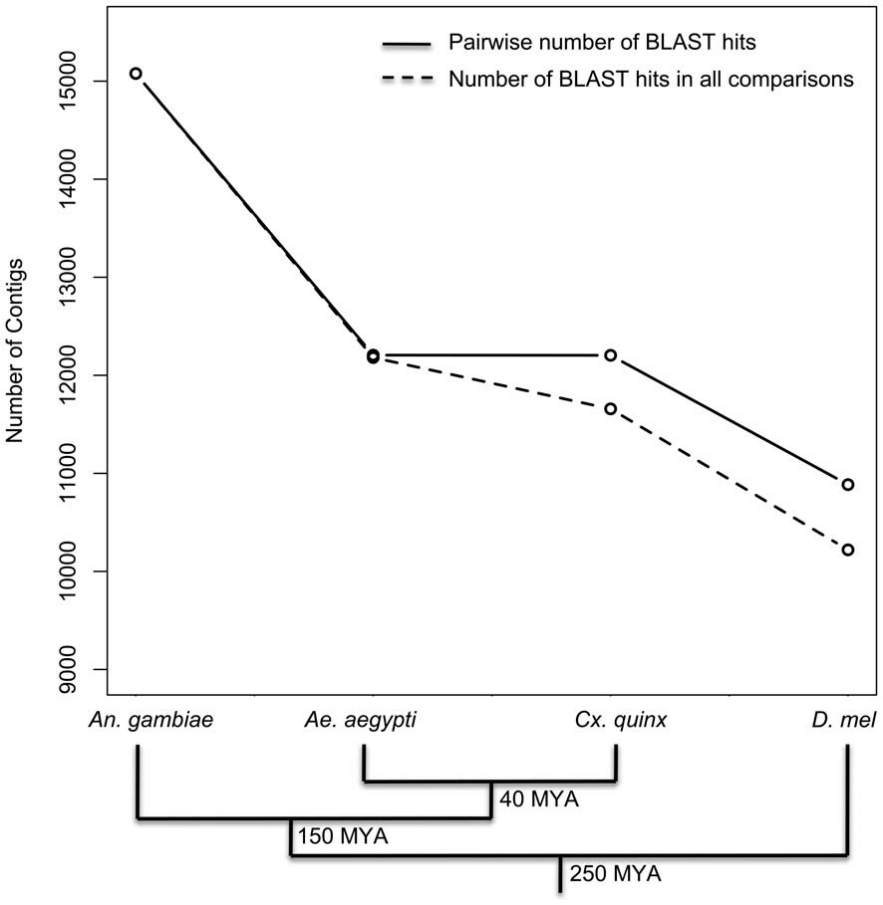
Homology with Dipteran transcriptomes decreases with increasing phylogentic difference. The number of *An. funestus* contigs with significant BLAST hits in pairwise comparisons to *An. gambiae, Ae. aegypti, C. quinquefasciatus* and *D. melanogaster* is plotted. Note that the y-axis only spans 9,000 to 15,000. The solid line indicates the total number of contigs with a significant BLAST hit in each comparison. The dashed line indicates the number of contigs with a significant BLAST hit in all comparisons as phylogenetic distance increases. The phylogenetic tree at the bottom of the panel depicts the evolutionary relationships between the Dipteran insects used in pairwise BLAST comparisons, with estimated divergence times (in millions of years) at each node (adapted from [53]).

In order to determine homology with available Dipteran sequences, we compared our contig set to predicted peptide sets extracted from four sequenced Dipteran genomes (*An. gambiae, Ae. aegypti, C. quinquefasciatus* and *D. melanogaster*) using the standalone BLASTX algorithm (e-value≤1×10^−6^) as well as to the *An. gambiae* genome with the TBLASTX (e-value≤1×10^−6^). We compared 15,527 *An. funestus* sequences to the closely related *An. gambiae* peptide set, finding 13,137 (84.6%) with significant similarity to an *An. gambiae* sequence, although this percentage may be slightly upwardly biased due to the usage of *An. gambiae* peptides during the clustering process in assembly step 3 (Materials and Methods). And while all contigs showed homology with at least one Dipteran transcriptome consistent with the selection process described above, a core set of 9,929 (63.9%) contigs showed significant matches in all four Dipteran transcriptomes (Figure 3). This high degree of sequence homology is consistent with previous observations of transcriptome conservation among these species [23]. Consistent with the expectation of increased divergence with increased phylogenetic distance, however, the number of contigs showing significant sequence similarity in pairwise comparisons between the *An. funestus* contig set and Dipteran transcriptomes decreased with increasing phylogenetic distance (Figure 3). It should be noted that the annotation process that produced the predicted peptide sets queried here were not independent since more recent annotations often train their gene model annotation pipeline on gene models from previously annotated genomes e.g. [17]. Highlighting the potentially limiting effect of this dependence, we found 2,360 contigs that showed no matches to the *An. gambiae* predicted peptide set but significant homology to the full *An. gambiae* genome sequence as well as other Dipteran sequences and sequences in functional domain databases. Although this discrepancy could be explained in part by differences between the BLAST algorithms employed in the two comparisons (TBLASTX for the genome versus BLASTX for the peptide set), it implies the presence of unannotated genes or transcribed units in the *An. gambiae* genome. A recent transcriptome profiling study also found many clusters of reads that mapped to unannotated regions of the *Ae. aegypti* genome [44] suggesting empirical validation of transcribed units using next-generation sequencing should be used to complement *in silico* gene prediction pipelines.

To best make direct comparisons between species, we searched for 1∶1 orthologous pairs between our *An. funestus* contigs and *An. gambiae* peptides, and putatively assigned 5,434 pairs using the standard reciprocal best-hit criteria. In a comparison between protein sequences of *An. gambiae*, *Ae. aegypti* and *D. melanogaster*, Waterhouse et al. [45] identified 4,951 1∶1∶1 orthologues and an additional 886 1∶1 *Anopheles*:*Aedes* orthologues, suggesting that our contig set harbors about 93.1% of the conserved, single-copy Dipteran orthologues. The median sequence similarity between 1∶1 orthologues identified here is 86.9% (σ_sim_ = 0.127), but 57.7% of our *An. funestus* contigs were shorter than their *An. gambiae* orthologue (mean proportion of *An. gambiae* transcript covered = 81.3%, σ_cov_ = 0.237), again suggesting that most of our transcripts are not full-length.

### Immune-system genes

When challenged by pathogens such as malaria parasites, mosquitoes mount a strong and effective innate immune response; so immune genes are of particular interest as potential points of exploitation for disruption of disease transmission [16]. To identify putative immune genes within our contig set, we downloaded a list of 414 *An. gambiae* genes annotated as immune genes in the ImmunoDB database [45]. We found significant sequence homology between 345 *An. funestus* contigs and 217 annotated *An. gambiae* immune genes. We also identified contigs with significant homology to 4 *An. gambiae* genes that have been functionally shown to be important in anti-malarial defense but that are not annotated in ImmunoDB: all three of the *APL1* genes (although we are unable to assign strict orthology) [46] and *LRIM1* [47].

Genes in the innate immune system can be split into four broad functional categories: recognition, signaling, regulation and effectors [45] [48]. We also included an additional category, ‘other’, to capture genes involved in other processes such as RNAi or autophagy that have been implicated in immunity. Based on significant BLAST matches to *An. gambiae* genes coding for recognition proteins including those annotated in ImmunoDB (e.g. Thioester-containing Proteins, Gram Negative Binding Proteins; n = 139) as well as the *APL1* paralogues and *LRIM1*, our *An. funestus* transcriptome contains 102 contigs that may function in pathogen recognition. We also recovered 33 contigs (33 in *An. gambiae*) putatively involved in immune signaling (e.g. *Cactus*, *Imd*), 108 putative immune regulatory contigs such as CLIP-domain serine proteases or Serine protease inhibitors (compared to 132 in *An. gambiae*), 33 putative effector genes (compared to 54 in *An. gambiae*; e.g. Cecropins, Lysozymes) and 69 contigs in the ‘other’ category putatively involved in RNAi (e.g. *Argonaute* and *Dicer*) and autophagy etc. (compared to 45 in *An. gambiae*). All matches between *An. funestus* contigs and *An. gambiae* immune genes are listed with their relevant immune annotations in Table S2.

**Figure 4:**
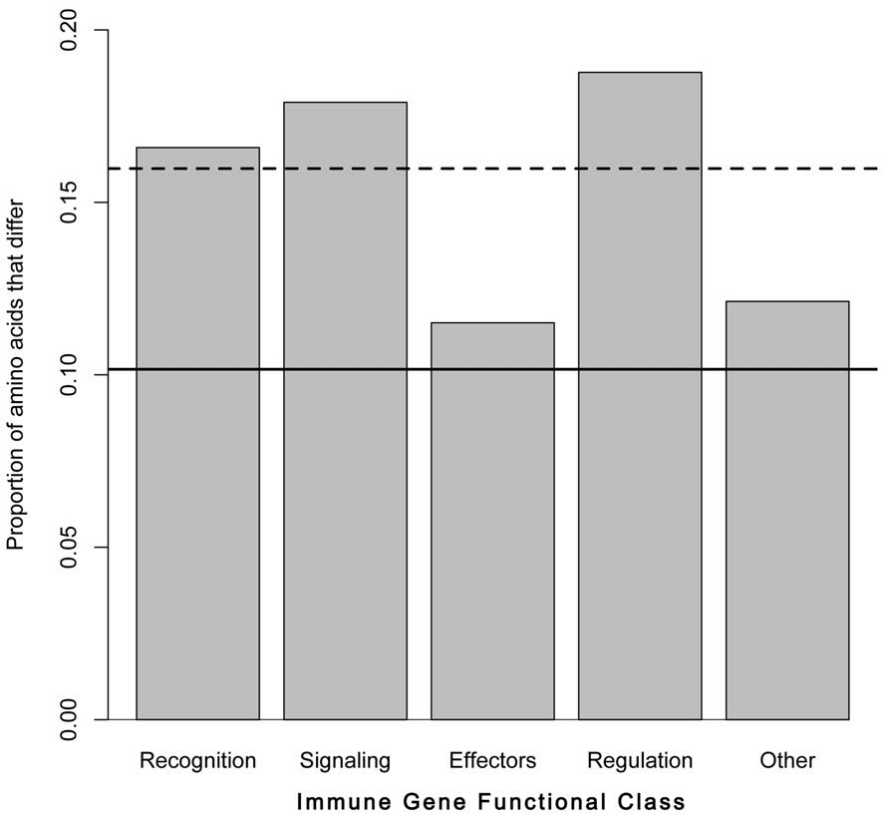
Variation in transcript divergence among immune gene functional classes. Protein sequence divergence was estimated as the proportion of aligned amino acids that differ between 1∶1 *An. funestus*:*An. gambiae* orthologues. As a class, immune gene orthologous pairs (dotted line indicates mean divergence between immune gene orthologues) are significantly more diverged than the transcriptome as a whole (solid line indicates mean divergence across the entire transcriptome; *p-*value = 4.8×10^−5^, Mann-Whitney U-test). The functional classes within the immune genes are not significantly different from each (*p-*values>0.05, pairwise Mann-Whitney U-tests).

Immune-system genes have been shown to be evolving at a faster rate than other genes in *Drosophila* and mosquitoes [45] [48]. We compared protein sequence divergence among 126 1∶1 orthologous pairs between *An. funestus* and *An. gambiae* with putative immune function to determine whether this observation holds true for our contig set. We found that orthologous pairs with putative immune function are significantly more diverged than the total set of all 1∶1 *An. gambiae*:*An. funestus* orthologous pairs (mean sequence differences for immune gene orthologues = 16.0%, n = 126, mean sequence differences among all orthologues = 10.2%, n = 4,975; *p* = 6.95×10^−9^, Mann-Whitney U-test). If we subdivide the analysis based on immune-system function, we find that regulatory proteins are most diverged (mean percent sequence differences = 18.77%), signaling proteins are second-most diverged (mean percent sequence differences = 17.90%), recognition proteins are third-most diverged (mean percent sequence differences = 16.59%) and effector proteins and proteins in the ‘other’ category are least diverged (mean percent sequence differences = 11.51% for effector, 12.13% for ‘other’), although only the regulatory and other categories are significantly different from each other (Figure 4; reg vs. other *p-*value = 0.0032, all other *p-*values range from 0.0566 to 0.8724, pairwise Mann-Whitney U-tests). These results suggest that the immune system genes of *An. funestus* are evolving in a fashion consistent with immune genes in other insects [45] [48]. Further studies dissecting the anti-pathogenic role each contig plays will greatly enhance our understanding of the mosquito immune system.

### Nucleotide diversity

Single nucleotide polymorphisms and short indels (collectively referred to as single/short nucleotide discrepancies, or SNDs) are very common in natural populations and provide valuable markers for genetic mapping as well as population genetic studies. We identified a set of 366,741 SNDs, suggesting approximately 1.95 SNDs exist in every 100 bp. The mean nucleotide diversity per contig was 0.019 per base before correction for variation in read depth and 0.024, (range of 0 to 0.163 per contig) after correction. This level of variation, particularly high coming from a colony, suggests that the colony may not have suffered the severe loss of genetic variation that could be expected after extended inbreeding. One explanation for this estimate is that the *An. funestus* colony was only recently established and thus too few generations of inbreeding have passed for the effect to be pronounced. Alternatively, our estimate of 0.024 per base may also be upwardly biased, however, by the presence of false-positives in our dataset, resulting from nucleotide mis-incorporation during polymerase chain reaction steps in template library preparation prior to sequencing. A previous Sanger-based re-sequencing study identified 494 SNPs from 20.5 kilobases of sequence (71.4% coding, with 303 SNPs mapping to the coding region), derived from a sample of 21 field and colonized specimens of *An. funestus* [49]. From this survey, they estimated a mean nucleotide diversity level of 0.007 [49], considerably lower than our estimate likely due to their smaller sample size. Estimates of nucleotide diversity in *An. gambiae*, a congeneric species with comparable generation time, geographical distribution and seasonality, are typically similar or perhaps slightly smaller than our estimate for this colony of *An. funestus* (e.g. [50]–[51]). The colony used in this study does not harbor the chromosomal rearrangements segregating in natural populations, but small and/or unknown inversions may be present and could play a role in preserving genetic variation at some loci. The genetic polymorphisms we have identified as segregating in this colony of *An. funestus* should be dispersed among all chromosomal arms and suggest that significant natural functional variation can still be found in the colony. Such variation may provide a valuable opportunity for future genetic mapping of phenotypes in the colony.

### Functional annotation of the whole transcriptome

To provide a biological foundation on which to begin to globally characterize the transcriptome, we sought to functionally annotate our contig set based on sequence homology to functionally annotated sequences in other species and identification of conserved protein domains. We identified 7,567 contigs that contained regions of significant homology to sequence in at least one database of protein domains (CDD, SMART, and Pfam). Comparisons to the KOG and GO databases provided putative functional information for 10,391 contigs. We were able to assign 3,506 unique GO annotations to 9,026 contigs (36,024 total matches), meaning that 58.1% of our total contigs have affinity to at least one GO term. These GO annotations are quite detailed and provide valuable information for specific contigs, but we were also interested in assigning contigs to broad functional categories that could be used to ask transcriptome-level biological questions. Therefore, we also found 28,781 associations between 119 generic GO-Slim annotations and 9,026 contigs. All annotation information is presented in Table S1, but only the GO-Slim annotations were used in the analyses described below, specifically focusing on 33 functional categories at the Cellular Component level, 39 categories at the Molecular Function level and 49 categories at the Biological Process level.

### Transcriptome divergence

**Figure 5:**
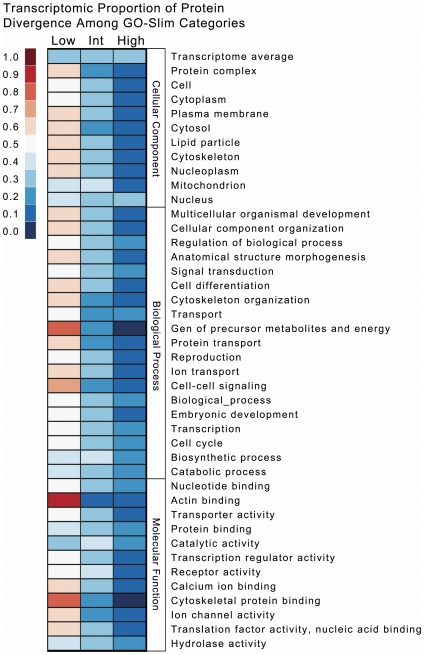
PROTEIN DIVERGENCE is unevenly distributed among GO-Slim categories. The heatplot shows proportion of 1∶1 orthologous pairs exhibiting Low, Intermediate and High protein divergence in GO-Slim functional categories. Protein divergence was estimated as the proportion of aligned amino acids that differed between the two orthologues and each orthologous pair was categorized as Low, Intermediate or High (Materials and Methods). Only categories whose proportion of each bin differed from expectations based on all orthologous pairs with a *p* value less than the Bonferroni-adjusted α of 5.26×10^−4^ are presented. The average expected proportions based on all orthologous pairs are presented at the top of the heatplot.

*An. funestus* and *An. gambiae*, estimated to have shared a common ancestor between 30 and 80 MYA [18], exhibit many ecological, behavioral and physiological differences. We examined levels of protein sequence divergence between 1∶1 orthologues to determine whether specific functional categories evolve at a rate that is different from the transcriptome as a whole. Of the functional categories tested, 10 Cellular Component categories, 20 Biological Process categories and 12 Molecular Function categories showed significant deviations from expected equal proportions of high, intermediate and low divergence categories at the Bonferroni-adjusted α level. Results for all categories are presented in Table S3, and significant results are presented in Figure 5. In general, significantly deviating categories tended to be enriched with lowly diverged orthologous pairs, indicating a high level of evolutionary conservation within these categories. While no categories were enriched with highly diverged pairs, we found a significant enrichment of intermediately diverged orthologous pairs localizing to the mitochondrion (Figure 5), as might be expected considering the known faster rate of evolution among genes associated with the mitochondrion. We also found significant enrichment of intermediately diverged pairs involved in lipid metabolic processes as well as in contigs with molecular functions involving catalytic activity and binding (Figure 5). A study of protein evolution among single copy orthologues across the phylogeny of the *D. melanogaster* species group identified 12 functional categories putatively under positive selection [52]. The categories identified here as enriched with intermediately diverged orthologous pairs are not among those 12, although immune genes identified outside of the GO-Slim analysis are significantly more diverged than the transcriptome [see above]. We note that our analysis probably has a strong bias toward detecting conserved sequences, since we limited our analysis to high confidence alignments and thus probably excluded highly diverged orthologous pairs. Nonetheless, our analysis offers the first glimpse into genome level patterns of protein evolution and a step towards a more comprehensive understanding of protein evolution in insect vectors.

### Expression profiling

Transcriptome sequencing in a non-model system makes it possible to conduct experiments to test hypotheses of differential expression between experimental treatments, for example. RNA-seq provides a powerful means of measuring gene-expression because the depth of sequence coverage of a transcript should be proportional to its expression level [4]. To demonstrate that a transcriptome assembled *de novo* can serve as a reference sequence for short-read mapping, we used the short read alignment program BWA to map three paired-end lanes of Illumina sequence to the final contig set. Of 101 million reads, approximately 51% of the reads were mapped uniquely to the transcriptome, while a fraction of a percent of the reads mapped to more than one location in the transcriptome. Interestingly, the remaining 49% of the reads were not successfully mapped, despite our somewhat liberal mapping criteria. It is possible that this rate of mapping success may reflect problems with this assembly, but a recent study mapping short sequence tags to the *Ae. aegypti* genome reported a comparable rate of mapping success [44] indicating that this rate is not likely to be an artifact of the *de novo* assembly process. Furthermore, we found that the profile of gene expression across contigs, as measured by reads per kilobase per million mapped reads (RPKM), adhered to the expected distribution with 95% of contigs having an RPKM value of 133.83 or less and extreme values that differ by three orders of magnitude (from 2.69 to 19,775.75). Therefore, we failed to find good evidence that transcriptomes assembled with our approach should not be used in short read mapping experiments.

### Concluding Remarks

Next generation short-read DNA sequencing has made it possible to explore genome-level questions in non-model organisms, regardless of their phylogenetic proximity to model species [3]. *An. funestus* is a primary vector of human malaria, but, as an experimental system, lags significantly in the availability of research data and scientific resources. To establish a genomic resource that will facilitate future genomic level studies in this species, we used the Illumina GAIIx sequencing platform and a novel assembly workflow to build the adult female *An. funestus* transcriptome. In doing so, we demonstrate the feasibility of Illumina-based transcriptome sequencing low cost ($6,300 in sequencing costs) and with the added value of obtaining quantitative expression and polymorphism data. We assembled a conservative and tractable set of 15,527 expressed *An*. *funestus* contigs, 5,434 of which could be identified as 1∶1 orthologues with the more distantly related species *An. gambiae*. We also identified contigs expressed in *An. funestus* that showed homology with unannotated regions of the *An. gambiae* genome, providing empirical evidence that these may be *bona fide* genes with orthologues that are currently unannotated in the *An*. *gambiae* genome. We identified almost 367,000 genome-wide polymorphisms segregating in our recently established *An. funestus* colony, and showed that, as expected, most of the *An. funestus* transcriptome is evolutionary constrained and is likely evolving under purifying selection. Our results highlight by example just some of the many questions that can be addressed using next-generation sequencing technology to explore the transcriptome of a non-model organism. We also supply essential tools for future genetic study of *An. funestus* and establish a novel *de novo* transcriptome assembly flow that should be applicable to any eukaryote.

## Supporting Information

Table S1 Contig Information Database. Excel spreadsheet containing information about each contig as well as all results from BLAST and functional domain database comparisons. Each row contains information for a single contig and each column contains a result for a specific analysis. NAs are inserted where there was either no result or the result did not apply to that contig. From left to right, the columns contain descriptive molecular information, BLAST results, functional annotation results and diversity and expression results.
(30.51 MB XLS)

Table S2 Putative An. funestus immune genes. Excel spreadsheet containing significant BLAST matches between *An. funestus* contigs and *An. gambiae* immune genes from ImmunoDB. BLAST e-values as well as relevant immunity annotations are presented.
(0.08 MB XLS)

Table S3 Protein Divergence GO-Slim Analysis. Excel spreadsheet containing number of contigs in each divergence bin and results of Χ2 analysis for each GO-Slim functional category.
(0.04 MB XLS)
